# Norecopa: A global knowledge base of resources for improving animal research and testing

**DOI:** 10.3389/fvets.2023.1119923

**Published:** 2023-02-02

**Authors:** Adrian J. Smith

**Affiliations:** Norecopa, c/o Norwegian Veterinary Institute, Ås, Norway

**Keywords:** animal research, testing, guidelines, replacement, reduction and refinement, PREPARE, quality

## Abstract

There are good ethical, legal and scientific reasons for ensuring that our use of animals in research and testing is limited to the lowest number of animals, and that those which are used are treated as humanely as possible, while at the same time providing reliable, reproducible and translatable data which is adequately reported. Unfortunately, there is widespread evidence that there is room for improvement in all these areas. This paper describes the Norecopa website, which offers links to global resources which can be used to resolve these issues. Much of the website content is linked to the PREPARE guidelines for planning any research or testing which appears to need animals. Attention to detail on all steps of the pathway from early planning to manuscript submission should lead to better science, improved animal welfare, and fewer health and safety accidents. This will also minimize the chances of manuscript rejection due to inadequate planning, avoiding a waste of human resources and animal lives.

## 1. Introduction

The implication that animal research can be improved may appear provocative to senior scientists. If their research has been funded, and the animal studies approved by an ethics committee and the relevant authorities, what more is to be gained?

Unfortunately, there is widespread evidence that animal research has suffered from poor reproducibility and translatability for many years [e.g., (1, 2)], and that the standard of reporting could be significantly improved [e.g., (3, 4)]. Better reporting depends upon better planning, for which there are now over 400 guidelines available worldwide, ^1^ in addition to the wealth of advice available at individual institutions.

The devil is often in the details in animal research and testing (5, 6). Norecopa has worked for the last 15 years to collect links to resources about the practical issues which decide the quality of this work. Many of these issues may appear obvious, but they regularly affect experiments which on paper look to be well-designed. Norecopa and coworkers have published the PREPARE guidelines (7) to offer scientists and animal care staff an overview of issues which should be considered when planning studies which appear to need animals, as aid to the advancement of the three Rs (Replacement, Reduction, Refinement) of Russell and Burch (8). PREPARE is based on experience gained from accrediting animal facilities, and from dialogue with scientists, animal technicians, regulators and veterinarians.

The PREPARE guidelines cover three main areas:

1. Formulation of the study
2. Dialogue between scientists and the animal facility
3. Quality control of the components in the study

**Figure 1:**
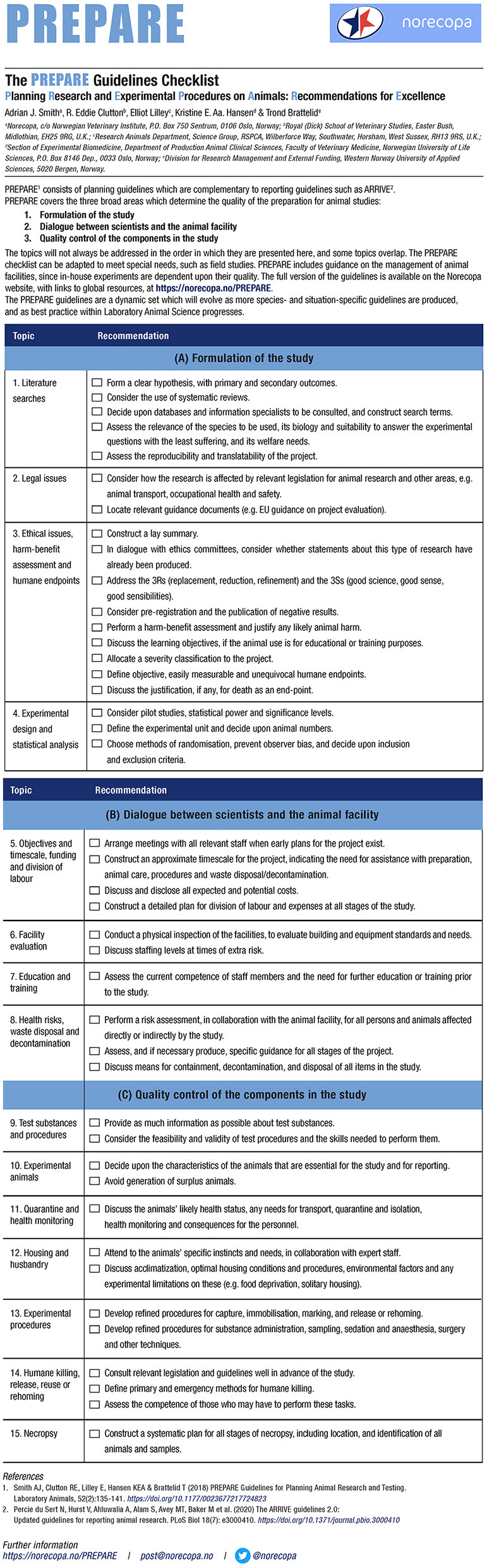
The PREPARE checklist (https://norecopa.no/PREPARE/prepare-checklist, accessed 8 January, 2022).

PREPARE consists of a checklist (see Figure 1, currently available in 34 languages) with 15 main topics covering these areas, and a website with links to more information, guidelines and scientific papers on each subject. The checklist is available in three different formats. The website is continuously updated, as new guidelines and relevant scientific papers are published. Many of these are announced first in Norecopa's newsletters.^2^

Some of the overarching aims behind the PREPARE guidelines are to promote:

1. The production of valid data (reflecting a true treatment effect, not artifacts caused, for example, by stress) from reproducible experiments. If the animal studies are designed to cast light on conditions in other species or humans, the results must be translatable to these.
2. Best possible animal welfare.
3. Attention to the health and safety of all those who are in any way affected by the animal studies, including other animals in the facility and visitors.
4. A good culture of care in the animal facility and research group.
5. Communication of best practice to other researchers and to the other stakeholders, including the general public.

A few brief examples from these areas are given below. Many more are cited on the PREPARE website.^3^

## 2. Valid, reproducible, and translatable data

There is ample evidence of both poor experimental design and reporting in the literature. Briefly, the major concerns (9) are:

- Lack of randomization and blinding.
- Low statistical power.
- Over-reliance on *p*-values, and *p*-value hacking.
- HARKing (hypothesizing after the results are known).
- Publication bias (under-reporting of negative or null results).

An overview of the concerns about the use of statistics has been published by Rowe (10).

What may be less clear to researchers is the need to modify their protocols to take into account the characteristics of the animals which they plan to use. The correct choice of species should be based upon knowledge of the hypothesis to be tested and the characteristics which a research animal must possess to be able to test the hypothesis, not solely upon which species have previously been used in the area. Among other things, physically smaller animals will in general have higher metabolic rates (11), which will affect the optimal dose rates.

Housing and husbandry conditions, and methods of handling and immobilization^4^,^5^ may all cause artifacts or stress which, in the worst case, can cause larger effects on the parameters to be measured than the experimental treatment itself [see also examples in (12)]. For example, laboratory animal diets have a far greater impact on experimental results than many realize (13). Åhlgren and Voikar (14) demonstrated how C57BL/6 mice purchased from two different suppliers showed behavioral differences which would affect the results of behavioral tests. Measurements of cardiac function are another example: Labitt et al. (15) identified changes in cardiac rhythm in mice for up to 6 mins following simple scruffing, an effect which could be eliminated by choosing a different method of grasping the skin on the neck.

## 3. Welfare

Adequate consideration of the welfare aspects of animal research must cover both animal and human welfare. Animal research has been dubbed “reversed veterinary medicine,” because it involves the procurement of healthy animals which are then treated in such a way that they become ill. This is the very opposite of the ideals imprinted in the education of animal care staff, and time must be set aside to inform all those who are skeptical about a new study of the benefits that will hopefully come from it, despite the very real likelihood of harm to the research animals themselves.

A good culture of care at an animal facility will help this process (see below), and will ensure that anyone who has reservations about a planned study has the confidence to speak their mind without fear of ridicule or reprisals.

Injections and blood samples which are relatively harmless in larger animals may cause significant distress in smaller ones. The stress of injections or oral gavaging can often be prevented by training animals for voluntary ingestion.^6^ The route and amount of blood sampling, which scientists may not be so focused upon, can dramatically affect the quality of the parameters to be measured in the samples and the welfare of the animals (16). Bleeding techniques may be evaluated against a number of subjective criteria based upon common sense:

- Is the blood vessel visible?
- Can the bleeding (including any internal blood loss) be easily stopped?
- Might the method damage surrounding tissue?
- Can the blood be collected quickly, to avoid artifacts in the samples due to variations in storage time before processing, or to mechanical damage caused by having to “milk” the vein to extract blood?

Considerable time may have to be spent designing studies which are minimally invasive and which cause the least effect on the animals' physiology. If humans are really the final target of a research programme, efforts should be made to see if animal studies can in fact be replaced by methods which use human materials.

Scientists and facility staff must both contribute to this process, not least because they are specialists in two different areas, both of which must be addressed when animal studies are planned. Scientists will naturally be mostly focused on the scientific hypotheses to be tested, and the more “mathematical” aspects of experimental design such as group size, experimental units and statistical analyses. Animal care staff will be more focused on practical issues related to space, equipment, staffing needs, competency and costs. They will also be more aware than the scientists of the potential sources of variability in the study that can be caused by intrinsic factors (e.g., genetic and microbial variation within the animals) and extrinsic factors such as the stress of transportation, social re-grouping, capture and handling, and environmental parameters (e.g., room temperature, humidity, and noise levels). Many of these are subtle and are still poorly understood or appreciated. While the potential causes of direct suffering in an experiment may be obvious, it may be more difficult to identify causes of what Russell and Burch called contingent suffering, i.e., pain and distress caused by other factors than the experiment itself, such as fighting when new social groups are established, or boredom in barren environments. A comprehensive slide deck describing the 3Rs has recently been published.^7^

## 4. Health and safety

Many research protocols, which appear scientifically sound on paper, present practical problems when preparations are made to implement them in an animal facility—or in the field. It is essential that maximum effort is made to avoid harm to anyone entering the area, or who may be exposed indirectly to harm from the experiment. This includes not only people but also other research animals. Many potentially harmful substances, such as radioactive isotopes, micro-organisms and carcinogenic drugs, are invisible and steps must be taken to ensure that they are properly contained. In addition to making sure that there are detailed protocols for their use, and emergency procedures in case someone is exposed to them, the facility must have adequate signage so that those entering the building are immediately made aware of the potential hazards and how to avoid them. Without adequate signs describing the presence of potential hazards, visitors to the facility, particularly if these are out of normal working hours, may accidentally be exposed.

Researchers who have worked with potential hazardous agents for many years will have developed their own routines for safe handling. They may not be aware of the need to inform animal care staff of these routines and, if necessary, to adjust them to the particular conditions in the animal facility, which may be very different from their laboratories. Likewise, their preferred methods of administration may not be realistic in the animals in question, necessitating a discussion. In many cases, the use of hazardous agents will lead to extra expense, for example to purchase additional protective clothing and to decontaminate the rooms afterwards. Discussions must include clarification of whom is to bear these costs.

Most significant accidents are caused by the presence of a number of smaller events which in themselves are relatively harmless, but which when they occur together trigger a significant event—the so-called Swiss Cheese effect (17).^8^ Threat and error management^9^ is an important part of running an animal facility, building in redundancy at critical steps, and including the establishment of contingency plans based upon a risk assessment (both of the facility and the research study).

Early dialogue between the research group and those who will be taking care of the animals is the clue to better science, better welfare and sufficient attention to health and safety. It is essential to clarify the responsibilities and costs at all stages of the study. These are, broadly speaking:

- Who is to perform which tasks?
- Who will be paying for equipment, procedures, training and staffing, over and above the standard functions of the facility?

They apply from the earliest preparations for a study all the way until the facility has been thoroughly decontaminated after the study and all material correctly disposed of.

These clarifications should be documented with an agreement signed by both parties. Such a document will greatly reduce the chances of research data being lost because one party had assumed that the other was going to collect them. A Master Plan should also be constructed, both for the study and for the animal facility itself, displaying the critical steps to be carried out and documentation of who has performed these. Standard Operating Procedures (SOPs) should be readily available for all of these steps, to aid harmonization. Discussions of all these stages of an experiment can greatly improve the quality of the study, as they tend to unearth potential challenges that might otherwise have been forgotten.

More advice on contingency plans and master plans is available on the Norecopa website.^10^ Loss of data can in the worst case result in inability to publish animal experiments, with a waste both of human resources and animal lives.

## 5. Culture of care

A Culture of Care is a commitment throughout a research facility to ensure mutual respect, so that everyone has the security and confidence to discuss potential concerns with an experiment or with the way in which the facility is managed. This commitment will automatically lead to an improvement in the scientific quality of the research, and the welfare of the animals in its care. The culture of care should also extend to transparency about the research, not least to the general public who in many cases are indirectly financing the research. A facility that has embraced this culture to its maximal extent will be a pleasant workplace producing high quality science from animals living in harmony with their surroundings.

Norecopa hosts the website of the International Culture of Care Network,^11^ which provides a forum to discuss ways in which this culture can be achieved and practical examples of resources and events that have been shown to work.

Since a caring environment empowers all employees to be able to raise their concerns, it will also embrace a Culture of Challenge (18), whereby staff look for acceptable methods of work, rather than being satisfied with what has been accepted.

The PREPARE guidelines emphasize the need for involvement of animal carers and technicians from the earliest stage of planning. There are a number of good reasons for this:

- they have a right to know about the aims of animal studies, and will be more motivated if they understand these,
- they, better than anyone else, know the possibilities (and limitations) inherent in the animal facility,
- they often possess a large range of practical skills and are good at lateral thinking from one species or project to another,
- they know the animals best,
- the animals know them best,
- lack of involvement creates anxiety, depression and opposition to animal research, as well as limiting creativity which might improve the experiments.

## 6. Communication of best practice

Improvements to protocols and facility management should be communicated quickly and widely so that others may benefit. In some cases this may entail writing a separate methodology paper to highlight the improvement. For example, the technique of blood sampling rodents from the saphenous vein was first described in one sentence in a research paper on a totally different subject (19), and it first became widely known and adopted after publication of a paper devoted entirely to it (20). These papers do not have to be published in journals with high impact factor, since the most important issue is to make them accessible as rapidly as possible to search engines.

Recently, a Refinement Wiki^12^ was created to allow even faster dissemination of such improvements, including more anecdotal accounts, with room for discussion.

Communication also entails honesty about failures or accidents. If others are to avoid the same mistakes, scientists must be prepared to report them. A service called CIRS-LAS (*Critical Incident Reporting System-Laboratory Animal Science*)^13^ has been designed for this purpose. Reports are submitted anonymously and published with a commentary on the CIRS-LAS website. The database can be searched for specific incidents.

## 7. The value of guidelines and checklists (standard operating procedures)

Although the 3R Guide database (see footnote 1) includes descriptions of over 400 guidelines, many more are still needed. In particular, species-specific guidelines need to be developed for housing and procedures. No better example of this is the need for more guidelines for fish, which are often treated as one group rather than separate species with very different needs.

Guidelines should be used as the basis for constructing checklists (or standard operating procedures as they are referred to in the realm of Good Laboratory Practice^14^) for ensuring the quality of each critical step of a study. In a busy research lab, there may be a tendency to look upon them as unnecessarily bureaucratic and time consuming, increasing the already substantial paperwork involved in animal research. On the contrary, just like a kitchen recipe (once it is written down), they save time, avoid errors, and ensure that the result is repeatable.

Checklists have many advantages. They are used extensively in the aviation industry to maintain their excellent safety record:

- They reduce risk of forgetting to carry out vital actions.
- They ensure that procedures are carried out in the correct sequence.
- They encourage cooperation and cross-checking between all players.
- They make sure that everyone is “on the same page.”

Norecopa has spent considerable resources on creating a website with links to global resources within animal research (both in the laboratory and in the field) and testing, animal welfare and related topics which can be helpful when planning, conducting and writing up animal studies. The website currently consists of roughly 9,000 pages, organized as one large searchable database. It includes a number of smaller databases such as NORINA (an inventory of ~2,800 alternatives or supplements to animal use in education and training),^15^ 3R Guide (a global collection of ~400 guidelines for animal research)^16^ and TextBase (~1,500 textbooks and other literature of relevance to this field).^17^ The website also contains information about, and links to, over 70 external databases which scientists may have use for.^18^

Access to these global resources has been facilitated by the overarching PREPARE Guidelines (7).

## 8. Concluding remarks

The pathway to better science consists of many steps, including some which are not the subject of this paper. Planning guidelines such as PREPARE (7) are, for example, complementary to reporting guidelines such as ARRIVE (4).

All too often, researchers are confronted at the submission stage with questions from reviewers which they are unable to answer, largely because these have not been addressed during the planning stage of the study. Conscientious use of planning guidelines, which act as an overarching checklist, will help researchers to identify and address these issues while it is still possible to act on them—for example to ensure that data which reviewers may later consider critical to the study is in fact recorded. Thorough planning will make it easier to perform adequate reporting, which in turn will greatly increase the likelihood of a manuscript being accepted for publication so that researchers will find that: “We ARRIVEd because we were PREPAREd.”

## Data availability statement

The original contributions presented in the study are included in the article/supplementary material, further inquiries can be directed to the corresponding author.

## Author contributions

The author confirms being the sole contributor of this work and has approved it for publication.

## Footnotes

^1^3R Guide. https://norecopa.no/3Rguide (accessed 8 January, 2022).

^2^https://norecopa.no/news/newsletters (accessed 8 January, 2022).

^3^https://norecopa.no/PREPARE (accessed 8 January, 2022).

^4^https://www.na3rsc.org/refined-mouse-handling (accessed 8 January, 2022).

^5^https://norecopa.no/prepare/12-housing-and-husbandry/12a/general-principles (accessed 8 January, 2022).

^6^https://www.ri.se/en/what-we-do/expertises/3r-focus-on-animal-welfare (accessed 8 January, 2022).

^7^https://norecopa.no/3Rs (accessed 8 January, 2022).

^8^https://en.wikipedia.org/wiki/Swiss_cheese_model (accessed January 8, 2022).

^9^https://www.montereynavyflyingclub.org/Safety%20Presentations/Intro%20to%20TEM%20Training%20v7a%20with%20VMax.pdf (Accessed January 8, 2022).

^10^https://norecopa.no/more-resources/master-plan-and-sops (accessed January 8, 2022).

^11^https://norecopa.no/coc (accessed January 8, 2022).

^12^https://wiki.norecopa.no/ (accessed January 8, 2022).

^13^https://www.cirs-las.de/ (accessed January 8, 2022).

^14^https://www.oecd.org/chemicalsafety/testing/good-laboratory-practiceglp.htm (accessed January 8, 2022).

^15^https://norecopa.no/NORINA (accessed January 8, 2022).

^16^https://norecopa.no/3RGuide (accessed January 8, 2022).

^17^https://norecopa.no/TextBase (accessed January 8, 2022).

^18^https://norecopa.no/search?fq=type:%22Databases%22&sort=name_s%20asc&q=*&facet.limit=125 (accessed January 8, 2022).
